# Near Misdiagnosis of Glioblastoma as Primary Central Nervous System Lymphoma

**DOI:** 10.14740/jocmr1846w

**Published:** 2014-05-22

**Authors:** Vijaya Raj Bhatt, Rajesh Shrestha, Nicole Shonka, R. Gregory Bociek

**Affiliations:** a Department of Internal Medicine, Division of Hematology-Oncology, University of Nebraska Medical Center, Omaha, NE, USA; b Department of Internal Medicine, Memorial Hospital of Rhode Island, Pawtucket, RI, USA

**Keywords:** Primary central nervous system lymphoma, Glioblastoma, Flow cytometry, Magnetic resonance imaging

## Abstract

Primary central nervous system (CNS) lymphoma, most frequently a diffuse large B-cell lymphoma, is a rare aggressive lymphoma confined to the CNS, thus requiring differentiation from other brain malignancies such as glioblastoma. Although stereotactic biopsy can confirm the diagnosis, this is invasive, not always feasible and can be inconclusive after steroid use. Hence, cranial magnetic resonance imaging (MRI) with contrast and cerebrospinal fluid analysis are frequently used to make a prompt diagnosis. We report a case of a woman with two brain masses who presented unique diagnostic challenge.

## Introduction

Primary central nervous system (CNS) lymphoma, most frequently a diffuse large B-cell lymphoma, is a rare aggressive lymphoma confined to the CNS, thus requiring differentiation from other brain malignancies such as glioblastoma. Although stereotactic biopsy can confirm the diagnosis, this is invasive, not always feasible and can be inconclusive after steroid use.

Cranial magnetic resonance imaging (MRI) and cerebrospinal fluid (CSF) analysis are commonly used diagnostic tests for primary CNS lymphoma [1].

## Case Report

**Figure 1:**
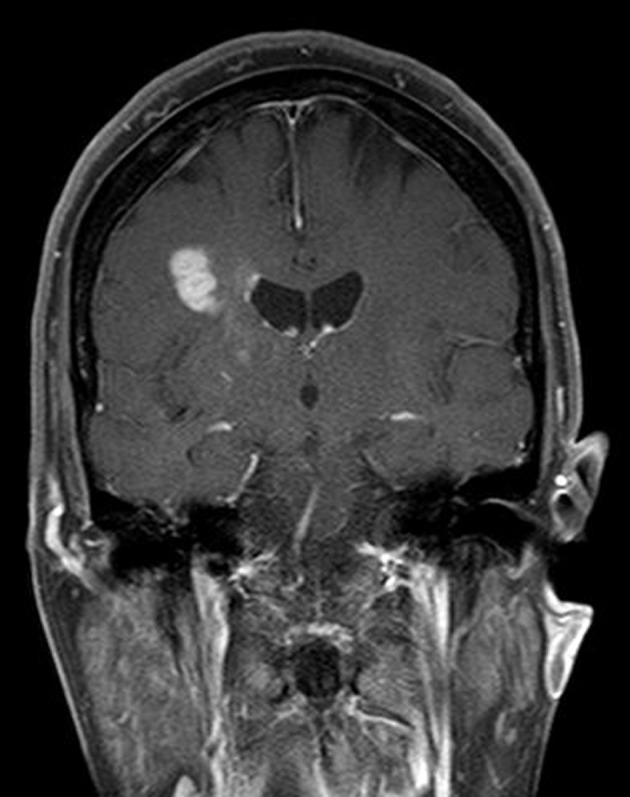
MRI of brain with and without gadolinium-based contrast demonstrating contrast enhancing lesion within the right posterior frontal lobe.

**Figure 2:**
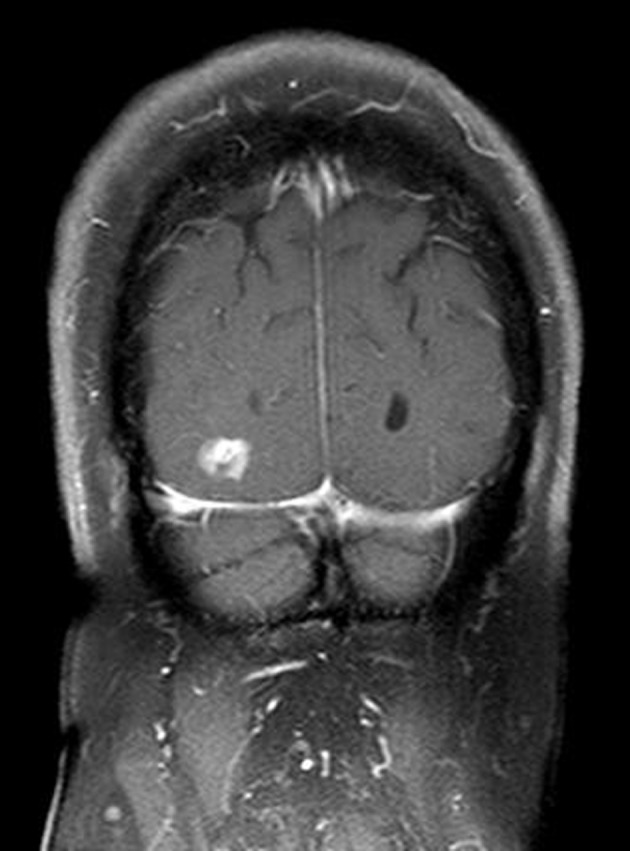
MRI of brain with and without gadolinium-based contrast demonstrating contrast enhancing lesion within the right occipital lobe.

A 74-year-old woman presented to our hospital with a 2-day history of left hemiparesis and dysarthria. She had left upper motor neuron facial palsy and mild left-sided hemiparesis. MRI of brain with and without gadolinium-based contrast revealed 2.5 cm enhancing lesion within the right posterior frontal lobe and 1.2 cm enhancing lesion within the right occipital lobe. These two lesions were abutting the right lateral ventricle, had little surrounding edema and mild diffusion restriction (Fig. 1, 2). The interpretation of the MRI was probable CNS lymphoma. CSF analysis revealed white blood cell count of 1/µL (67% lymphocytes), red blood cell count of < 3,000/µL, protein of 51 mg/dL and glucose of 103 mg/dL (blood glucose of 165 mg/dL). Flow cytometry of CSF revealed a population of B-cells expressing CD19, CD20, CD24, CD38 and high-density kappa light chains at 4.5% of lymphocytes. Computed tomography scan of chest, abdomen and pelvis with contrast did not reveal any lymphadenopathy or hepatosplenomegaly. Thus, primary CNS lymphoma was considered the most likely diagnosis. A bone marrow biopsy performed for staging revealed hypercellularity (50%) with a population of mature B-cells expressing CD19, CD20, CD24, CD38 and high-density kappa light chains at 26% of lymphocytes, consistent with B-cell non-Hodgkin lymphoma (NHL). Within the regional laboratory computer system, a 2010 bone marrow report showing a similar finding was found. On questioning further, it was determined that the patient had been evaluated by a hematologist-oncologist in the past. Medical records obtained from that physician’s office revealed a diagnosis of B-cell NHL involving only bone marrow. A hematopathology review of the CSF cytospin revealed small lymphocytes without any large lymphocytes. A hematopathology review of peripheral smear demonstrated occasional small lymphocytes consistent with circulating lymphoma cells. With this information, it was felt that the clonal cells in the CSF sample represented peripheral blood contamination. A subsequent stereotactic biopsy of the brain mass confirmed the diagnosis of glioblastoma.

## Discussion

National Comprehensive Cancer Network guidelines on primary CNS lymphoma recommend CSF analysis (cytology and flow cytometry, gene rearrangements optional) for diagnosis in patients with characteristic MRI findings. Brain biopsy is recommended if CSF analysis and ophthalmology evaluation with ocular biopsy of suspicious lesion are not diagnostic [2]. This approach may avoid unnecessary morbidity associated with brain biopsy in a subset of patients.

Characteristic MRI findings of primary CNS lymphoma include homogenous and strong enhancement, periventricular location, single or multiple lesions, without necrosis and with relatively less edema [1, 3]. Recent studies have highlighted that B-cell primary CNS lymphoma can also be differentiated from glioblastoma based on fractional anisotropy and apparent diffusion coefficient on diffusion tensor imaging [4] and the intratumoral susceptibility signals on susceptibility weighted imaging [5], both of which are new MRI techniques. However, as illustrated by this report, glioblastoma can closely mimic the MRI characteristics of primary CNS lymphoma.

Although traditional CSF studies (cell count, protein and glucose) have low sensitivity and specificity for CNS lymphoma, CSF flow cytometry and immunoglobulin heavy chain rearrangement have moderate specificity. CSF cytology and less commonly available, CSF antithrombin III and micro RNA analysis have high specificity [3]. CSF analysis including CSF flow cytometry is also used in clinical practice to rule out CNS involvement from NHL. In a study among newly diagnosed aggressive NHL, positive CSF flow cytometry was associated with significantly higher risk of CNS relapse and poorer outcomes [6]. Although CSF flow cytometry is useful in the diagnosis of primary CNS lymphoma, as highlighted by this case, peripheral blood contamination can be the source of lymphoma cells. This is particularly important since traumatic spinal tap occurs in up to 20% of cases [7]. In conclusion, a diagnosis of primary CNS lymphoma should be made after evaluation for any possible systemic NHL, exclusion of circulating lymphoma cells by hematopathologic review of peripheral blood smear or peripheral blood flow cytometry, morphological assessment of lymphoma cells in CSF cytospin (which can differentiate large lymphocytes seen in the majority of primary CNS lymphoma from contaminating small lymphocytes seen in monoclonal B-cell lymphocytosis or other NHL) and lastly brain biopsy if there is any doubt.
